# A Randomized Controlled Trial Comparing Ambu AuraGain and i-gel in Young Pediatric Patients

**DOI:** 10.3390/jcm8081235

**Published:** 2019-08-16

**Authors:** Ha-Jung Kim, Hee-Sun Park, Soo-Young Kim, Young-Jin Ro, Hong-Seuk Yang, Won Uk Koh

**Affiliations:** 1 Department of Anesthesiology and Pain Medicine, University of Ulsan College of Medicine, Asan Medical Center, Seoul 05505, Korea; 2 Department of Anesthesiology and Pain Medicine, Sun Medical Center, Daejeon 34811, Korea

**Keywords:** Ambu AuraGain, i-gel, pediatric patients, supraglottic airway device

## Abstract

Supraglottic airway devices have been increasingly used because of their several advantages. Previous studies showed that the small-sized i-gel provides effective ventilation for young pediatric patients; however, few studies have reported the use of AuraGain in these patients. Herein, we compared the clinical performance of AuraGain and i-gel in young pediatric patients aged between 6 months and 6 years old and weighing 5–20 kg, who were scheduled to undergo extremity surgery under general anesthesia. In total, 68 patients were enrolled and randomly allocated into two groups: AuraGain group and i-gel group. The primary outcome was the requirement of additional airway maneuvers. We also analyzed insertion parameters, fiberoptic bronchoscopic view, oropharyngeal leak pressure, and peri-operative adverse effects. Compared with the AuraGain group, the i-gel group required more additional airway maneuvers during the placement of the device and maintenance of ventilation. The fiberoptic view was better in the AuraGain group than in the i-gel group. However, the oropharyngeal leak pressure was higher in the i-gel group. AuraGain might be a better choice over i-gel considering the requirement of additional airway maneuvers. However, when a higher oropharyngeal leak pressure is required, the i-gel is more beneficial than AuraGain.

## 1. Introduction

Supraglottic airway devices form a group of airway devices that are inserted into the pharynx for ventilation. Since their introduction into medical practice, supraglottic airway devices have proven safe and efficient in various settings [1]. In addition, they have several advantages over endotracheal tubes. Insertion of supraglottic airway devices provides smooth induction of anesthesia without hemodynamic instability [2], and their insertion is easier and faster than intubation, especially for those unfamiliar with endotracheal intubation [3,4]. Although controversial, some previous studies have reported that supraglottic airway devices reduce the incidence of postoperative airway complications [5,6,7,8]. Given these advantages, supraglottic airway devices have been increasingly used worldwide.

i-gel^®^ (Intersurgical, Wokingham, Berkshire, UK)—the representative second-generation supraglottic airway device—is constructed using a thermoplastic elastomer to fit the perilaryngeal structure for effective airway management. In addition, with a bite block and lumen for the gastric tube, i-gel is optimized for safe airway management. A small-sized i-gel is available for children and provides satisfactory airway under general anesthesia [9,10]. However, a study suggested that i-gel had a tendency of sliding out from the appropriate position in pediatric patients and that it should be used cautiously [11].

Ambu^®^ AuraGainTM (Ambu, Ballerup, Denmark), a relatively novel supraglottic airway device, has been introduced recently. AuraGain has features different from those of i-gel. AuraGain has an inflatable cuff and a curved body. In addition, because the airway tube of AuraGain is wide, it can be used as a conduit for tracheal intubation. However, only a limited number of studies have been conducted on the performance of AuraGain in pediatric patients, and only few studies have reported that AuraGain is a safe and effective device for young children [12].

In this study, we aimed to compare the clinical performance of AuraGain and i-gel in young children weighing under 20 kg. The primary outcome was the requirement of additional airway maneuvers, including the adjustment of head/neck position, adjustment of device insertion depth, and taping of the device. We also analyzed (a) insertion time and success rate at first attempt, (b) fiberoptic bronchoscopic view, (c) oropharyngeal leak pressure and the change in oropharyngeal leak pressure during 10 min, (d) the degree of ease of gastric tube insertion, and (e) peri-operative adverse effects.

## 2. Materials and Methods

This parallel group, randomized controlled trial was conducted in accordance with the Declaration of Helsinki, and the protocol was approved by the Institutional Review Board of Asan Medical Center (2017–1101; date of approval, 18 September 2017). Written informed consent was obtained from the parents of all patients. We registered this study on ClinicalTrials.gov (NCT03294226).

### 2.1. Participants

In this study, we included patients who met the following criteria: (1) American Society of Anesthesiologists physical status 1–3, (2) age between 6 months and 6 years old, (3) weight between 5 and 20 kg, and (4) scheduled to undergo upper/lower extremity surgery under general anesthesia at a tertiary center in Seoul, Korea. Patients with known difficult airway, high risk of aspiration including those with gastrointestinal stenosis or stricture, and symptoms or signs of upper respiratory infection were excluded. Patients who were scheduled to undergo day-surgery and those who refused to be included in this study were also excluded. All the included patients were randomly allocated into two groups, namely, the AuraGain and i-gel groups. In this study, since the body weight of the patients ranged from 5 kg to 20 kg, two sizes of devices were used. Since the device size, which is decided by body weight, could jeopardize the result of this study, we performed stratified randomization considering body weight as the stratification factor. We divided the patients into two strata according to their body weight (i.e., less than 10 kg and over 10 kg), and different device sizes (i.e., 1.5 and 2, respectively) of AuraGain and i-gel were randomly assigned at a 1:1 ratio in each stratum. The allocation details were concealed in an opaque sealed envelope, which was opened only before the insertion of the device in the operating room.

### 2.2. Anesthesia

After the patients arrived in the operating room, they were positioned supine with their head resting on a donut-shaped pillow. They were pre-oxygenated with 100% oxygen, and propofol (2 mg·kg^−1^) mixed with lidocaine was injected intravenously. When the eyelash reflex was lost, the patient’s lung was mask-ventilated with 8 vol% sevoflurane in 100% oxygen for 3 min. Neuromuscular blocking agents were not used. After the patient reached an adequate anesthetic depth, which was verified by the absence of motor and cardiovascular responses to the jaw thrust maneuver, the assigned supraglottic airway device was inserted. The supraglottic airway device was lubricated with lidocaine jelly during insertion. The size of the inserted device was chosen according to the manufacturer’s recommendation (i-gel: size 1.5 for 5–9.9 kg and size 2 for 10–20 kg; AuraGain: size 1.5 for 5–9.9 kg and size 2 for 10–20 kg). In the case of AuraGain, the cuff was inflated and the intracuff pressure measured using a manometer was set at 30–40 cmH_2_O. Anesthesia during surgery was maintained using 2.5–3% sevoflurane in 50% nitrous oxide and 50% oxygen passing through the inserted device. Volume-controlled ventilation was applied to all patients, and the tidal volume was chosen on the basis of the patient’s body weight. Respiratory rate was controlled to maintain normocapnia. When self-respiration recovered intraoperatively, the pressure support mode was applied. In these patients, the pressure and respiratory rate were adjusted according to minute ventilation to maintain normocapnia. At the end of the surgery, the inhalation anesthetics were discontinued, and the lung was ventilated with 100% oxygen again. After the recovery of self-respiration, the device was removed. All the procedures were performed by two anesthesiologists who were also study authors. The anesthesiologists were skilled and vastly experienced at inserting supraglottic airway devices.

### 2.3. Definition of Device Failure

We defined device failure as three failed insertion attempts of a device. A failed insertion attempt was defined as the inability to insert the assigned device or insufficient ventilation (<6 mL·kg^−1^) or high airway pressure (peak airway pressure > 30 cmH_2_O) to achieve sufficient ventilation despite the manipulation of the device, including the adjustment of head/neck position and changing the insertion depth. After an event of device failure, the device was removed from the mouth and endotracheal intubation was performed with 0.6 mg·kg^−1^ of rocuronium bromide.

### 2.4. Measurements

The primary outcome was the requirement of additional airway maneuvers to provide sufficient ventilation, including the adjustment of head/neck position, adjustment of device insertion depth, and taping to prevent the device from sliding out during placement and maintenance in young children weighing under 20 kg. Sufficient ventilation was determined according to the following criteria: no audible leakage with manual ventilation of 20 cmH_2_O and bilateral symmetric chest movements. The requirement of taping was judged on the basis of whether there was movement or rotation of the device from the initial position and whether the movement or rotation caused insufficient ventilation. Insertion time was defined as the time from the moment the mask was taken away from the patient’s face to the moment stable capnography was traced on the monitor with the presence of sufficient ventilation. The fiberoptic view, which was used for evaluating the anatomic alignment of the supraglottic airway device to the larynx, was obtained using a fiberoptic bronchoscope inserted through the supraglottic airway device. The best view observed at 1 cm above the tip of the ventilating orifice was graded according to the Brimacombe grading scale: Grade 1, only larynx seen; Grade 2, larynx and epiglottis posterior surface seen; Grade 3, larynx and epiglottis tip or anterior surface seen—visual obstruction of the epiglottis to the larynx <50%; Grade 4, epiglottis down-folded, and its anterior surface seen—visual obstruction of the epiglottis to the larynx >50%; and Grade 5, epiglottis down-folded, and the larynx cannot be seen directly [13]. Oropharyngeal leak pressure was measured by closing the expiratory valve of the circle system to 30 cmH_2_O at a fixed gas flow of 3 L·min^−1^ and by recording the airway pressure at which equilibrium was reached [14]. The digital readout of the anesthesia machine defined the oropharyngeal leak pressure. Oropharyngeal leak pressure was measured at 1 min after the successful insertion of the device, and this was repeated at 10 min after the insertion of the device. Ease of gastric tube insertion scored by the anesthesiologist and peri-operative adverse effects, including aspiration of gastric fluid, bronchospasm without desaturation, transient desaturation, dental/tongue/lip trauma, and blood staining on the removed device, were also recorded.

### 2.5. Statistics

To determine the sample size, we conducted a pilot study on nine patients. In the pilot study, half of the patients in the i-gel group and 20% of those in the AuraGain group required additional airway maneuvers. The calculated sample size with α = 0.1 and power = 80% by using a two-sample, two-sided equality test was 30 patients in each group. Considering dropouts, we decided to assign 34 patients into each group. All data were recorded using a standardized case report form, and were analyzed using PASW Statistics for Windows, Version 18.0 (SPSS Inc., Chicago, IL, USA). Categorical, continuous, and ordinal data were compared using Fisher’s exact test, Student’s t-test, and Mann–Whitney U test, respectively. We not only analyzed the overall data without considering the device size but also analyzed the data for each device size. A *p* value less than 0.05 was considered statistically significant.

## 3. Results

**Figure 1:**
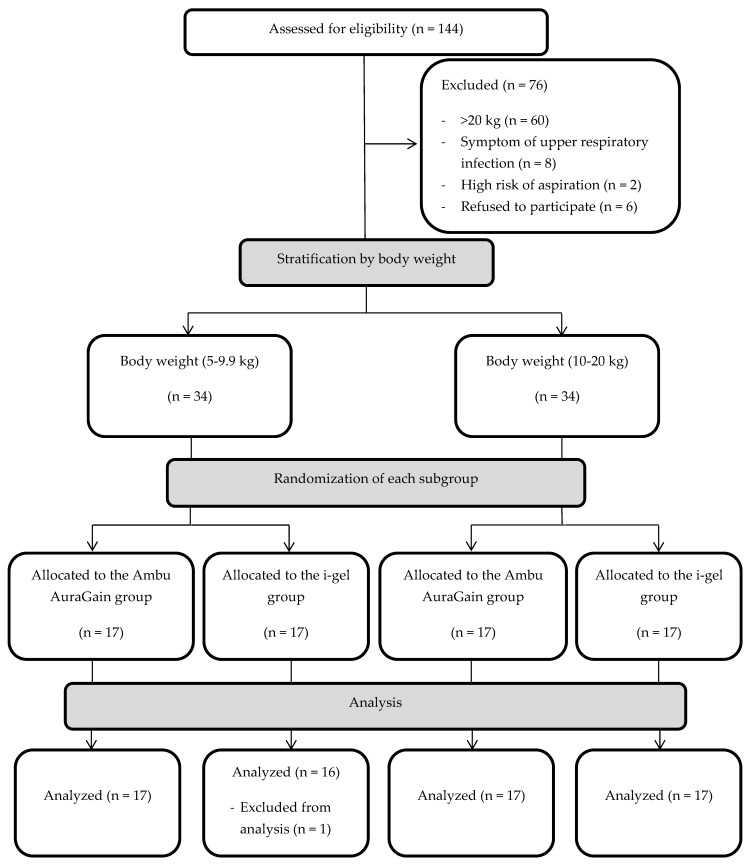
Flow diagram for the process of study screening and assignment of the study patients.

In total, 144 children aged between 6 months and 6 years old and weighing under 20 kg, who were scheduled to undergo elective upper or lower limb surgery at our institution from September 2017 to March 2018, were screened for eligibility. Among them, 70 patients were found ineligible for this study because of the following reasons: 60 patients were heavier than 20 kg, two had a high risk of aspiration, and eight had a symptom of upper respiratory tract infection. Seventy-four patients who qualified for this study were asked for informed consent. The parents of six patients refused inclusion in the study. The remaining 68 patients who agreed to participate were allocated into two groups, namely, the AuraGain and i-gel groups. However, one patient with laryngospasm during device insertion in the i-gel group was excluded from the analysis, since device failure was determined, and an endotracheal tube was inserted (Figure 1).

All the variables were expressed as mean (± standard deviation) or number (%). Table 1 shows the demographic data. No significant difference was observed between the two groups except in age. Despite the randomization, the mean age of the AuraGain group was higher than that of the i-gel group.

The primary outcome is shown in Table 2. During the placement of the device, the i-gel group required much more additional airway maneuvers, including the adjustment of head/neck position, adjustment of device insertion depth, or taping of the device, than did the AuraGain group (*p* < 0.001). In detail, the adjustment of device insertion depth and taping were required more frequently in the i-gel group during the placement of the supraglottic airway device, while the adjustment of head/neck position showed no significant difference. Subgroup analysis based on device size revealed results similar to the overall results. During the maintenance of ventilation, the i-gel group required significantly more additional maneuvers than did the AuraGain group (*p* = 0.033). In detail, only the adjustment of device insertion depth showed a significant difference. In the subgroup analysis based on device size, we found the difference only in the requirement of adjustment of device insertion depth in the size 2 subgroup (*p* = 0.039).

Secondary outcome variables are presented in Table 3. Most outcome variables did not differ between the two groups, and only the fiberoptic bronchoscopic view and oropharyngeal leak pressure were significantly different. The fiberoptic bronchoscopic view was better in the AuraGain group (*p* = 0.008), while i-gel showed significantly higher oropharyngeal leak pressures than did the AuraGain at 1 min and 10 min (*p* < 0.001). In addition, we compared the fiberoptic bronchoscopic view between groups in each stratum: while there was no difference between groups of size 1.5 devices (AuraGain group, 2.4 ± 1.1; i-gel group, 2.7 ± 1.1; *p* = 0.404), AuraGain group showed significantly better fiberoptic bronchoscopic view compared to that of i-gel group in patients using size 2 devices. (AuraGain group 1.6 ± 0.8; i-gel group, 2.9 ± 1.3; *p* = 0.008). However, in adverse effects, no difference was observed between the two groups.

## 4. Discussion

Our findings demonstrated that AuraGain has an advantage over i-gel because it requires fewer additional airway maneuvers for effective ventilation in young children. However, i-gel also provides adequate ventilation and higher oropharyngeal leak pressure than does AuraGain.

The result that patients in the i-gel group required more adjustment of the device or head/neck position of the patient during placement is consistent with that of a previous study, even though the previous study compared the clinical performance of i-gel with that of Ambu AuraOnce [15]. This study also differs from the previous study in terms of the age of the included patients. We included only young pediatric patients weighing under 20 kg, whereas Theiler et al. included 0- to 17-year-old patients weighing 5–50 kg, and only six children weighed 5–9.9 kg [15]. The pediatric airway is not a small version of the adult airway, and it has different characteristics from those of the adult airway [16]. In particular, in infants and young pediatric patients, the disparities are more remarkable than they are in older children [17]. Nevertheless, the two studies showed similar results. Theiler et al. explained the result on the basis of the differences in the shape of the ventilation tube [15]. AuraGain, the second-generation supraglottic airway device in the Aura family, has a 90-degree curved body similar to that of Ambu AuraOnce. The curved shape of AuraGain might contribute to its better fit into the laryngeal anatomy in contrast to the i-gel, which has a relatively straight body [18]. In addition, the volume of the inflatable cuff of AuraGain was adjusted according to the patient, which enabled better alignment to the larynx. Other studies have also reported the spontaneous dislodgement of i-gel, and authors have speculated that it might be due to the wider mask portion of i-gel [19,20]. As i-gel has a tendency to slide out from the mouth, taping the device following depth adjustment was frequently required. In addition, we found that 30% of patients in the i-gel group needed manipulation of the device or head/neck position even during the surgery. This result might be partially related to the younger age of patients in the i-gel group despite the randomization. The age range of the children included in this study was within the period of tooth development. Although it did not reach statistical significance, patients with molar teeth were more common in the AuraGain group, with a higher average age than in the i-gel group. However, as shown in the subgroup analysis, AuraGain seems to have a better fixation than does i-gel, wherein AuraGain required less additional airway maneuvers in patients with no tooth or incisor teeth alone. Therefore, when i-gel is used in young children, more vigilant monitoring would be required during the peri-operative period.

Recently, the Difficult Airway Society recommended trying to insert a supraglottic airway device as a rescue method in case of unanticipated difficult tracheal intubation in children [21]. Furthermore, if endotracheal intubation is absolutely necessary, they suggest considering endotracheal intubation via the inserted supraglottic airway device [21]. Both devices used in this study were designed to have a capability to act as a conduit for intubation and they have relatively larger lumens to ease intubation. The fiberoptic bronchoscopic view reflects the alignment of the supraglottic airway device with the larynx, and it provides a hint about successful intubation using the supraglottic airway device. In this study, down-folding of the epiglottis corresponding to Brimacombe score grades 4 and 5 was observed in nine patients in the i-gel group, but only in two patients in the AuraGain group. Our sub-analysis showed that regardless of size, AuraGain provides a better fiberoptic view than does i-gel. Although both devices provided effective ventilation even with epiglottic down-folding, AuraGain was considered a better conduit for intubation. Jagannathan et al. also reported AuraGain’s excellence as a conduit based on the good fiberoptic view [12]. Therefore, in case of intubation through the supraglottic airway device for young pediatric patients, AuraGain may be a better choice over i-gel.

However, i-gel has an advantage over AuraGain when considering oropharyngeal leak pressure. This result is consistent with those of previous studies. A clinical trial that compared the clinical performance of i-gel and AuraOnce showed significantly higher oropharyngeal leak pressure in the i-gel group [15]. A meta-analysis also identified that oropharyngeal leak pressure was higher with i-gel than with other laryngeal mask airway families in children [22]. We speculated that the relatively large-sized mask might contribute to this result. Having a higher oropharyngeal leak pressure results in better sealing of the hypopharynx, and this might be advantageous in clinical settings requiring increased airway pressure. The large-sized mask of i-gel is a disadvantage with respect to dislodgement but an advantage with respect to oropharyngeal leak pressure.

Many previous studies on pediatric patients demonstrated that both devices are inserted successfully and that insertion of the devices takes only 20 s or less [12,20,23]. In our study, in almost all patients, both devices were inserted at the first attempt except in one patient in the i-gel group. In addition, the placement of the devices required only 15 s in the AuraGain group and 16 s in the i-gel group, without any discernible differences between the two groups. Insertion of i-gel without an inflatable cuff helped save more time, but the final time was similar because additional airway interventions were needed during i-gel insertion. The high success rate at the first attempt and short insertion time confirm the benefits of using supraglottic airway devices in young children.

Gastric access through the gastric channel was successful in many patients. The presence of a channel for gastric tube placement is a feature of the second-generation supraglottic airway devices, and this reduces the risk of aspiration when removing the air insufflated during bag-mask ventilation and expanded with the use of nitrous oxide [24]. Although it did not reach statistical significance, gastric access tended to be easier in the i-gel group than in the AuraGain group. The straighter body of i-gel might facilitate easier gastric tube insertion.

The occurrence of peri-operative adverse effects in the present study was similar to or greater than that in other studies. In previous randomized controlled trials, the authors reported no incidence of airway reflex spasm in patients using i-gel or Ambu AuraGain [12,25]. This disparity might be because of the difference in the condition of removal of the device. Alzahem et al. and Jagannathan et al. removed the device under deep anesthesia [12,25], while we removed the device when the airway reflexes were restored. Although debatable, some anesthesiologists argue that fewer respiratory complications occur after removing the device under deep anesthesia [9].

The current study has some limitations. First, some patients included in this study were too young to provide reliable answers; thus, we could not evaluate post-operative airway complications including sore throat and hoarseness. Second, this study was single-blinded. The patients were blinded, but the experimenters who evaluated the outcome variables were not blinded. However, device insertion was performed by only two skilled anesthesiologists to lessen interindividual differences, and the variables were measured simultaneously during device insertion; hence, this limitation was inevitable.

## 5. Conclusions

In conclusion, our study identified that Ambu AuraGain required less additional airway maneuvers for successful ventilation than did i-gel during the placement and maintenance of the device in young pediatric patients. AuraGain might also be more beneficial than i-gel as a conduit for tracheal intubation. However, i-gel would be more advantageous when a high oropharyngeal leak pressure is required.

## Figures and Tables

**Table 1:** Baseline characteristics of the children undergoing general anesthesia with Ambu AuraGain and i-gel.

	Ambu AuraGain (*n* = 34)	i-gel (*n* = 33)
Age (months)	23.5 (±17.8)	15.6 (±11.5)
Males/females	21/13 (61.8/38.2)	17/16 (51.5/48.5)
Height (cm)	83.7 (±13.3)	77.9 (±10.5)
Weight (kg)	11.6 (±3.3)	10.5 (±2.4)
Body mass index (kg·m^−2^)	16.4 (±1.9)	17.3 (±2.1)
Operation time (min)	49.1 (±34.6)	50.2 (±34.4)
Anesthesia time (min)	83.1 (±38.2)	80.4 (±38.0)
Presence of molars		
No	17 (50.0)	24 (72.7)
Yes	17 (50.0)	9 (27.3)
Type of surgery		
Hand surgery, number (%)	19 (55.9)	23 (69.7)
Arm surgery, number (%)	0 (0)	3 (9.1)
Hip surgery, number (%)	0 (0)	2 (6.1)
Foot surgery, number (%)	15 (44.1)	5 (15.2)

Data are presented as mean (± standard deviation) and number (%).

**Table 2:** Comparison of the requirement of additional airway maneuvers for both devices.

Variable	AuraGain (*n* = 34)	i-gel (*n* = 33)	*p* Value	Size 1.5			Size 2		
				AuraGain (*n* = 17)	i-gel (*n* = 16)	*p* Value	AuraGain (*n* = 17)	i-gel (*n* = 17)	*p* Value
Additional airway maneuvers during placement									
P/D/T†	4 (11.8)	25 (75.8)	<0.001	3 (17.6)	12 (75.0)	0.002	1 (5.9)	13 (76.5)	<0.001
P‡	3 (8.8)	8 (24.2)	0.109	2 (11.8)	1 (6.3)	>0.999	1 (5.9)	7 (41.2)	0.039
D⁕	1 (2.9)	23 (69.7)	<0.001	1 (5.9)	11 (68.8)	<0.001	0 (0.0)	12 (70.6)	<0.001
T⁑	3 (8.8)	22 (66.7)	<0.001	3 (17.6)	11 (68.8)	0.005	0 (0.0)	11 (64.7)	<0.001
Additional airway maneuvers during maintenance									
P/D/T†	3 (8.8)	10 (30.3)	0.033	1 (5.9)	3 (18.8)	0.335	2 (11.8)	7 (41.2)	0.118
P‡	3 (8.8)	7 (21.2)	0.186	1 (5.9)	2 (12.5)	0.601	2 (11.8)	5 (29.4)	0.398
D⁕	1 (2.9)	9 (27.3)	0.006	0 (0.0)	2 (12.5)	0.227	1 (5.9)	7 (41.2)	0.039
T⁑	0 (0.0)	0 (0.0)	-	0 (0.0)	0 (0.0)	-	0 (0.0)	0 (0.0)	-

Data are presented as number (%). P/D/T†, adjustment of head/neck position or device insertion depth or taping; P‡, adjustment of head/neck position; D⁕, adjustment of device insertion depth; T⁑, taping.

**Table 3:** Comparison of the clinical performances of both devices other than the requirement of additional airway maneuvers.

Variable	AuraGain (*n* = 34)	i-gel (*n* = 33)	*p* Value
Insertion time (s)	13.3 (±3.7)	13.1 (±4.9)	0.677
Success rate at first attempt	34 (100)	32 (97)	0.493
Oropharyngeal leak pressure			
at 1 min (cmH_2_O)	18.6 (±4.2)	23.3 (±4.6)	<0.001
at 10 min (cmH_2_O)	18.9 (±3.5)	23.3 (±4.0)	<0.001
Absolute value of the change in oropharyngeal leak pressure (1 to 10 min)	2.7 (±2.7)	3.3 (±3.3)	0.384
Fiberoptic bronchoscopic view 1/2/3/4/5	12/12/8/1/1 (35.3/35.3/23.5/2.9/2.9)	6/7/11/6/3 (18.2/21.2/33.3/18.2/9.1)	0.008
Ease of gastric tube insertion 1/2/3	27/5/2 (79.4/14.7/5.9)	29/4/0 (87.9/12.1/0.0)	0.500
Adverse effect			
Aspiration of gastric fluid	0 (0)	0 (0)	-
Bronchospasm with/without desaturation	2 (5.9)	5 (15.2)	0.259
Transient desaturation	1 (2.9)	3 (9.1)	0.356
Dental/tongue/lip trauma	0 (0)	0 (0)	-
Blood staining on the removed device	5 (14.7)	1 (3.0)	0.197

Data are presented as mean (± standard deviation) and number (%).
